# Special Issue: Biopolymers in Drug Delivery and Regenerative Medicine

**DOI:** 10.3390/molecules26030568

**Published:** 2021-01-22

**Authors:** Ricardo Starbird-Perez, Pasquale Del Gaudio, Carlos A. García-González

**Affiliations:** 1 Centro de Investigación y de Servicios Químicos y Microbiológicos (CEQIATEC), School of Chemistry, Instituto Tecnológico de Costa Rica, 159-7050 Cartago, Costa Rica; 2 Centro de Investigación en Ciencia e Ingeniería de Materiales (CICIMA), Universidad de Costa Rica, 11501-2060 San José, Costa Rica; 3 Department of Pharmacy, University of Salerno, Via Giovanni Paolo II 132, I-84084 Fisciano, Italy; 4 Department of Pharmacology, Pharmacy and Pharmaceutical Technology, I+D Farma Group (GI-1645), Faculty of Pharmacy and Health Research Institute of Santiago de Compostela (IDIS), Universidade de Santiago de Compostela, E-15782 Santiago de Compostela, Spain

Biopolymers and biocomposites have emerged as promising pathways to develop novel materials and substrates for biomedical applications. Moreover, the availability, processability, and low toxicity of biopolymers encourage their use in drug delivery and regenerative medicine. The engineering of biopolymer-based materials for their structuration at the nano- and microscale along with their chemical properties allows the design of advanced formulations used as carriers for drug products and as cell scaffolding materials. Finally, combination products including—or based on—biopolymers for controlled drug release offer a powerful solution to improve the tissue integration and biological response of these materials.

In this Special Issue, original research and review articles ranging from the chemical synthesis and characterization of modified biopolymers to their processing in different morphologies and hierarchical structures and in vitro and in vivo evaluation have been gathered together with the aim of contributing to the progress in the biomedicine field.

Recent findings on the applications and processing of biopolymers in the drug delivery area are presented. In a review article, Osorio et al. [1] described different polymer-based drug delivery systems, including polysaccharides and derived polysaccharides, used for the specific case of colorectal cancer treatments. In another biomedical application, Goel et al. carried out a preclinical study (New Zealand White rabbits model) to test the use of keratose, an oxidized form of keratin, as a drug excipient to control the release of paclitaxel, an anti-proliferative drug, from coated biomedical devices (angioplasty balloons) for the treatment of peripheral arterial disease [2]. The benefits of the presence of this biopolymer, in terms of minimizing the adverse impact of peripheral vascular motion on drug retention and reaching a favorable biological response, were assessed. From the processing point of view, López-Iglesias and coworkers [3] introduced a novel production strategy of solid lipid microparticles using a solvent-free technique, the so-called Particles from Gas-Saturated Solutions (PGSS^®^) process. Artificial neural networks and fuzzy logic tools were used to model the process in terms of temperature, pressure and nozzle diameter to obtain optimized microparticulate systems. The obtained lipid microparticles can be of a potential interest in several biomedical, food and environmental applications. In an alternative processing approach, a comprehensive review by Auriemma et al. presents a description of the theoretical and practical aspects behind the production of different polysaccharide-based hydrogel particles with a special focus on the prilling technology in tandem with several ageing and drying techniques [4]. The value of these techniques is related to its versatility and scalability. Moreover, the gel drying method is a critical step that makes it possible to obtain carriers with specific morphology and inner structure, which may have effects on the release properties. Particularly, the processing of dry polysaccharide-based gels, in the form of cryogels and aerogels, is an emerging field for drug delivery, and European initiatives (e.g., the European Commission-funded AERoGELS CA18125 COST Action) are currently ongoing, with the aim of displaying the full potential of these nanostructured materials for biomedical purposes [5,6]. In this regard, an alternative approach based on the synthesis starch cryogels from enzymatically modified starch combined with lyophilization has been reported by Boccia and coworkers [7]. The chemical nature plays a crucial role in the ability of the prepared cryogels from modified starch to act as drug carriers with promising applications for wound healing and for certain specific administration routes. Additionally, in an experimental application from Pantić et al. [8], tablet-shaped pectin aerogels and pectin aerogels coated with an external layer of chitosan were tested as carriers for oral administration of curcumin, being able to modify the release profile of this drug. Results showed that uncoated pectin aerogel provided a prompt curcumin release, whereas the multilayer biopolymer-based aerogel resulted in a carrier providing a prolonged release of the drug during 24 h.

Advantageous uses of biopolymers as cell scaffolds or for extracellular stimulation were presented in this Special Issue for the development of diverse strategies and implant systems in the regenerative medicine field. In a review article by Alvarado-Hidalgo et al. [9], the recent uses and fabrication methods of biopolymers for extracellular biochemical stimulation of cells as biomimetic 3D scaffold systems were presented. The potential modulation of the extracellular environment through mechanical, electrical, and biochemical stimulation was introduced, with a particular focus on biophysical and biochemical cues of cells that drive their molecular reprogramming. An example of this concept was presented in the research article from Ramírez Sánchez et al. [10]. A smart conductive composite material was processed using κ-carrageenan as a doping agent to improve its electrical properties. The obtained material was electroactive and allowed the release of dexamethasone by electrochemical stimulation, providing a stable system to be used in bioelectronic applications. Finally, cellulose phosphate aerogels for biomedical uses were developed by Schimper et al. [11] through an environmentally friendly methodology based on the conversion of biomass into functional materials. The obtained derivatized cellulose aerogels can be beneficial for the design of dual-porous cell scaffolding materials with interconnected mesopores and micron-size pores.

Therefore, the objective of the Special Issue on “Biopolymers in Drug Delivery and Regenerative Medicine”—to provide the most recent advances on biopolymer research for biomedical applications, particularly in regenerative medicine and drug delivery—has been extensively achieved. 

## References

[1] Osorio M., Martinez E., Naranjo T.W., Castro-Herazo C. (2020). Recent Advances in Polymer Nanomaterials for Drug Delivery of Adjuvants in Colorectal Cancer Treatment: A Scientific-Technological Analysis and Review. Molecules.

[2] Goel E., Erwin M., Cawthon C.V., Schaff C., Fedor N., Rayl T., Wilson O., Christians U., Register T.C., Geary R.L. (2020). Pre-Clinical Investigation of Keratose as an Excipient of Drug Coated Balloons. Molecules.

[3] López-Iglesias C., López E.R., Fernández J., Landin M., García-González C.A. (2020). Modeling of the Production of Lipid Microparticles Using PGSS® Technique. Molecules.

[4] Auriemma G., Russo P., Del Gaudio P., García-González C.A., Landin M., Aquino R.P. (2020). Technologies and Formulation Design of Polysaccharide-Based Hydrogels for Drug Delivery. Molecules.

[5] AERoGELS CA18125 COST Action Website. https://cost-aerogels.eu/.

[6] García-González C.A., Budtova T., Durães L., Erkey C., Del Gaudio P., Gurikov P., Koebel M.M., Liebner F., Neagu M., Smirnova I. (2019). An Opinion Paper on Aerogels for Biomedical and Environmental Applications. Molecules.

[7] Boccia A.C., Scavia G., Schizzi I., Conzatti L. (2020). Biobased Cryogels from Enzymatically Oxidized Starch: Functionalized Materials as Carriers of Active Molecules. Molecules.

[8] Pantić M., Horvat G., Knez Ž., Novak Z. (2020). Preparation and Characterization of Chitosan-Coated Pectin Aerogels: Curcumin Case Study. Molecules.

[9] Alvarado-Hidalgo F., Ramírez-Sánchez K., Starbird-Perez R. (2020). Smart Porous Multi-Stimulus Polysaccharide-Based Biomaterials for Tissue Engineering. Molecules.

[10] Ramírez-Sánchez K., Ledezma-Espinoza A., Sánchez-Kopper A., Avendaño-Soto E., Prado M., Starbird-Perez R. (2020). Polysaccharide κ-Carrageenan as Doping Agent in Conductive Coatings for Electrochemical Controlled Release of Dexamethasone at Therapeutic Doses. Molecules.

[11] Schimper C.B., Pachschwoell P.S., Hettegger H., Neouze M.-A., Nedelec J.-M., Wendland M., Rosenau T., Liebner F. (2020). Aerogels from Cellulose Phosphates of Low Degree of Substitution: A TBAF·H2O/DMSO Based Approach. Molecules.

